# Statement of Eng’s Widow

**Published:** 1874-03

**Authors:** 


*Statement of Eng's widow, made to the Commission at Mount Airy.*—The paralytic stroke from which Chang had suffered occurred about three years ago, when he was at sea, seven days out from Liverpool. He had been intemperate for some time previously, and had been drinking hard on board the vessel, being frequently intoxicated. He had never had mania-a-potu. Even when he was drunk, Eng was not affected. Two of his children had died; one from phthisis, the other apparently from apoplexy. Eng had lost five children: one each from phthisis, diptheria, and dysentery; one from the effects of a burn, and one still-born.

Chang died Saturday, January 17. He had had a cough since the preceding Monday night. It was dry, with scanty, frothy sputum, and no pain. Left lung probably involved; slight dulness on corresponding side. On Thursday, January 15, his skin was acting freely, and for that reason Dr. Hollingsworth ordered him not to venture out; but, in spite of that prohibition, he went as usual to Eng's. At the time of his arrival he had little cough and no expectoration, but loud bronchial rales were plainly heard by those around him.

When Eng saw his wife, after learning that Chang was dead, he said, "I am dying," but did not speak of his brother's death. He soon afterwards expressed a desire to defecate, and this continued for half an hour. He rubbed his upper extremities, raised them restlessly, and complained of a choking sensation. The only notice he took of Chang was to move him nearer. His last words were, "May the Lord have mercy on my soul!"

